# The Impact of Marital History on Late-Life Loneliness and Social Participation: A U.S.-China Comparison

**DOI:** 10.1093/geroni/igaf122.1314

**Published:** 2025-12-31

**Authors:** Leping Wang, Yang (Claire) Yang

**Affiliations:** Vanderbilt University, Nashville, Tennessee, United States; The University of North Carolina at Chapel Hill, Chapel Hill, North Carolina, United States

## Abstract

In this paper, we use HRS and CHARLS data to investigate the relationship between marital history over the life course and loneliness and social participation in late life among older adults aged 65 and over across the U.S. and China, and the gender differences therein. We show that in the U.S., marital trajectories are more heterogeneous than in China, and non-normative marital transitions are more prevalent. U.S. and Chinese older adults exhibit compositional differences in social participation. Chinese older adults predominately meet up with friends, whereas more U.S. older adults volunteer or participate in organized group meetings. Marital history is associated with loneliness and social participation differently by country and by gender. Off-time marital transition is associated with worse socio-emotional well-being than on-time transition, represented by the higher likelihood of loneliness among Chinese widowed prematurely. On-time transition, featured by mid-life divorce in the U.S., is otherwise linked to less loneliness. U.S. Women widowed prematurely and staying lifelong single have more active social participation than men, whereas never-married Chinese women are less active in social participation than men. Our findings reveal the impact of marital histories, including the states and transitions, on the socio-emotional well-being of older adults from two interconnected themes: the life course framework and the normative transition theory. We also highlight the importance of studying the holistic marital history (rather than current marital status) and its socio-psychological consequences over the life course, with particular attention to non-normative marital transitions that standardized measures of marital status tend to overlook.

